# Linkage Disequilibrium-Based Inference of Genome Homology and Chromosomal Rearrangements Between Species

**DOI:** 10.1534/g3.120.401090

**Published:** 2020-05-20

**Authors:** Daniel Jordan de Abreu Santos, Gregório Miguel Ferreira de Camargo, Diercles Francisco Cardoso, Marcos Eli Buzanskas, Rusbel Raul Aspilcueta-Borquis, Naudin Alejandro Hurtado-Lugo, Francisco Ribeiro de Araújo Neto, Lúcia Galvão de Albuquerque, Li Ma, Humberto Tonhati

**Affiliations:** * Departamento de Zootecnia, Jaboticabal-SP,; ^‡^ Departamento de Ciências Exatas Universidade Estadual Paulista (Unesp), Brazil,; ^†^ Department of Animal and Avian Sciences, University of Maryland, College Park, College Park, Maryland, and; ^§^ Instituto Federal Goiano, Campus Rio Verde, Rio Verde GO, Brazil

**Keywords:** Buffalo, Cattle, SNP, Haplotype Diversity, Genotype Imputation, Genome Assembly, Chromosome Evolution

## Abstract

The aim of this study was to analyze the genomic homology between cattle (*Bos taurus*) and buffaloes (*Bubalus bubalis*) and to propose a rearrangement of the buffalo genome through linkage disequilibrium analyses of buffalo SNP markers referenced in the cattle genome assembly and also compare it to the buffalo genome assembly. A panel of bovine SNPs (single nucleotide polymorphisms) was used for hierarchical, non-hierarchical and admixture cluster analyses. Thus, the linkage disequilibrium information between markers of a specific panel of buffalo was used to infer chromosomal rearrangement. Haplotype diversity and imputation accuracy of the submetacentric chromosomes were also analyzed. The genomic homology between the species enabled us to use the bovine genome assembly to recreate a buffalo genomic reference by rearranging the submetacentric chromosomes. The centromere of the submetacentric chromosomes exhibited high linkage disequilibrium and low haplotype diversity. It allowed hypothesizing about chromosome evolution. It indicated that buffalo submetacentric chromosomes are a centric fusion of ancestral acrocentric chromosomes. The chronology of fusions was also suggested. Moreover, a linear regression between buffalo and cattle rearranged assembly and the imputation accuracy indicated that the rearrangement of the chromosomes was adequate. When using the bovine reference genome assembly, the rearrangement of the buffalo submetacentric chromosomes could be done by SNP BTA (chromosome of *Bos taurus*) calculations: shorter BTA (shorter arm of buffalo chromosome) was given as [(shorter BTA length – SNP position in shorter BTA)] and larger BTA length as [shorter BTA length + (larger BTA length – SNP position in larger BTA)]. Finally, the proposed linkage disequilibrium-based method can be applied to elucidate other chromosomal rearrangement events in other species with the possibility of better understanding the evolutionary relationship between their genomes.

The *Bubalus bubalis* species spread out across the world from Asia where it was domesticated (Nagarajan *et al.* 2015; Zhang *et al.* 2016); however, the relationship between the species and other bovids is still not clear, especially when it concerns genetics. The Bovini tribe, from the Bovinae subfamily, includes *Bos* (cattle) and *Bubalus* (buffalo) genera, as well as *Bison*, *Syncerus* and *Pseudoryx* (Vaughan 1986; Hassanin and Douzery 1999). The differences between these genera are mainly due to domesticity, geographical origin, number of chromosomes and some morphological peculiarities. Despite the diversity between the genera, similarities between buffaloes and cattle are observed not only because of the external morphology, but also due to the domestication by humans. The genus *Bubalus* contains five species of buffalo, among which *Bubalus bubalis* (water buffalo) stands out due to productive aptitude (Tanaka *et al.* 1996; Borguese 2005). The water buffalo can be further divided into two main groups, the river buffalo (2n = 50) represented by the Mediterranean, Murrah and Jafarabadi breeds, across others, and the swamp buffalo (*Bubalus bubalis carabanensis*) (2n = 48), represented by Carabao (Iannuzzi 1994; Pérez-Pardal *et al.* 2018). Both groups are used for traction in the Asian continent; however, the river buffalo has a better productive capacity, being destined for meat and milk production (Roth and Myers 2004).

The chromosomal homology between buffaloes and cattle was verified by cytogenetic probes and by sequence-level (Low *et al.* 2019). All the cattle autosomes are acrocentric; buffaloes have five submetacentric autosomes and the others are acrocentric. The buffaloes’ five submetacentric chromosomes correspond to centric fusions of the *Bos taurus* autosomes (BTAs) 1;27, 2;23, 8;19, 5;28 and 16;29 (Iannuzzi *et al.* 2003; Michelizzi *et al.* 2010). Some studies have compared the chromosomal structures of bovines and buffaloes to reveal the structural homologies and chromosomal aberrations (Pauciullo *et al.* 2014; Iannuzzi *et al.* 2015; Stafuzza *et al.* 2015) using the techniques of fluorescence and hybrid maps.

The homology between buffalo and bovine species is especially important because it allows transferring the developed genomic technologies between the two species. Genotyping panels developed for cattle are being used in buffaloes (Michelizzi *et al.* 2011; Wu *et al.* 2013; Borquis *et al.* 2014), and a specific panel, developed by Affymetrix for the buffalo species has markers annotated in the cattle genome (de Camargo *et al.* 2015; Iamartino *et al.* 2017). It was done because the buffalo genome was just recently sequenced and assembled using PacBio technology (Williams *et al.* 2017; Low *et al.* 2019), while the cattle genome was assembled since 2009 (Zimin *et al.* 2009). The assembly of the cattle genome reference is more complete than the buffalo genome and it has been extensively studied in four independent projects (Bovine Genome Sequencing; Analysis Consortium 2009; Zimin *et al.* 2009; Koren *et al.* 2018; and ARS-UCD1.2, https://www.ncbi.nlm.nih.gov/assembly/GCA_002263795.2), while the buffalo genome reference by two recent projects (Thibaud-Nissen *et al*. 2019; Low *et al.* 2019). The last two cattle assemblies, as well as the first buffalo genome were assembled using PacBio technology, and the buffalo genome showed higher quality compared to goat and human assemblies (Low *et al.* 2019). The recent UMD_CASPUR_WB_2.0 used Illumina HiSeq technology and it is available in scaffolds (Thibaud-Nissen 2019). These latest advances in the buffalo genome assemblies have allowed discoveries of SNPs (single nucleotide polymorphisms) as well as other specific genomic structures to the buffalo (Li *et al.* 2019; Surya *et al.* 2019).

There are few genomic studies in buffaloes when compared to cattle. Since cattle and buffaloes are close on the evolution scale; cheap and fast solutions of transferring recent genomic technologies between the species can be realized. Thus, the first aim of this study was to verify the genomic homology across buffaloes (Murrah breed - *B. bubalis*) and others bovids through DNA variations (polymorphisms) using a cattle SNP panel. We verified the genetic homology between buffaloes and cattle, and rearranged a bovine reference genome for the five buffalo submetacentric chromosomes based on SNP-linkage disequilibrium. A specific buffalo panel was used to assess the chromosome rearrangement in buffaloes via linkage disequilibrium comparing it to a cattle reference genome and to the buffalo genome assembly reference. This study also generated hypotheses regarding the evolution of the buffalo submetacentric chromosomes.

## Material and Methods

### Ethics statement

The present study did not require approval of the ethics committee. The biological material used for DNA extraction was previously collected and stored (de Camargo *et al.* 2015).

### Animals and genotyping data

The buffalo genotyping was done with DNA extracted from hair follicle samples of animals from the Milk-Recording Program. The data set consisted of 349 Murrah water buffaloes (37 sires and 312 dams) genotyped with Affymetrix Axiom Buffalo Genotyping Array (90k-123,040 SNPs) and 363 (16 sires and 347 dams) genotyped with the Illumina BovineHD BeadChip (770k). The genotypes for the Affymetrix Axiom Buffalo Genotyping assay were obtained using a custom cluster file, where all buffalo samples were clustered together. A total of 214 animals were genotyped with both panels. All samples had a call rate higher than 0.90.

The genetic relationship of buffaloes with other bovids was investigated using the data available for download from the WIDDE database (Sempéré *et al.* 2015). The database has information of *Bos taurus taurus* (42 Angus and 60 Holstein), *Bos taurus indicus* (27 Gir and 41 Nelore), and 6 *Bubalus depressicornis* genotyped with BovineHD BeadChip (770k), 20 *Bos javanicus* and 20 Boran animals (*Bos taurus indicus*) genotyped with Illumina Bovine SNP50 BeadChip (50k).

### Genetic clusters

This analysis only considered samples of buffaloes genotyped with the Illumina cattle panel along with other bovids. Two clustering methods were used. The non-hierarchical method used the principal component analysis (PCA), obtained from the genomic relationship matrix of all animals using the PLINK software (Purcell *et al.* 2007). A call rate greater than 0.95 and minor allele frequency (MAF) of at least 0.02 were adopted for genotype quality control. The hierarchical method used Ward approach for Jaccard similarity coefficient using the R statistical software (R Core Team 2013). In order to calculate the similarity across populations, we used the alleles on each population, instead of individual genotypes (like in PCA). Since the global minor allele of each *locus* defined as the one with the highest frequency in most of the species, three MAF limit values for all genotype dataset: 0.1, 0,3 and 0.5 (regular limit), were also tested. Lower limits mean greater restriction to the markers.

### Introgression analyses

The same set of markers used in PCA was used for this analysis. To facilitate the graphical view, a subset of 62 animals out of the 363 *B. bubalis* was used. The ADMIXTURE 1.21 software (Alexander *et al.* 2009) was used to evaluate the proportion of introgression among the different populations, considering k number of clusters, with k ranging from 2 to 8.

### Linkage disequilibrium and chromosomal rearrangement

For this analysis, only the genotyped buffaloes were used. A total of 46,378 and 13,142 SNPs of the Affymetrix and Illumina panels respectively were considered (call rate> 0.95, MAF> 5% and p-value for HWE >10^−6^). Only the autosomal markers and markers with known position in the conventional bovine reference genome (UMD3.1) were used in the analysis. The LD (linkage disequilibrium) between the pairs of markers was calculated by the r^2^ statistic using the PLINK software (Purcell *et al.* 2007). Only r^2^> 0.2 between all the markers (intra and inter-chromosome) were used to proceed with the chromosome clustering and rearrangement. Chromosome, by bovine reference map, with markers that had a high correlation with markers in other chromosomes were rearranged by ordering the parts (beginning or end) highly correlated (number of markers in high LD) in a single chromosome. The values of the new coordinates were obtained by adjusting the bovine reference to the sum and to the orientation of the chromosome structures that merged, and the starting point was always from the end of the short arm of the buffalo chromosome.

With the buffalo specific panel, a scan performed on the UAO_WB_1 assembly for rearranged chromosomes to verify the constancy of the LD level along these chromosomes. It would indicate recent fusion (high LD) or deleted (low LD) regions in buffalo genome. It also allowed comparisons of the level of LD in centromeric and telomeric regions. For this, we used r^2^ estimates adjusted for windows with size of 4 Mb that slide every 1 Mb. The estimates of r^2^ were adjusted using the Hill and Weir (1988) decay function given by the following formula:

$$E(r^{2})=\left[\frac{10+C^{}}{\left(2+C\right)\left(11+C\right)}\right]\left[1+\frac{\left(3+C\right)\left(12+12C+C^{2}\right)}{n\left(2+C\right)(11+C)}\right]$$

It is a nonlinear function with a single coefficient for physical distance, C, which is the least-squares estimate for 4Nec (Ne = effective population size and c is the recombination fraction between sites) per base pair distance between markers, and n is the sample size. The analyzes were performed using the statistical R program (R Core Team 2013).

### Genome assemblies and misassembly signature

Due to the fact that buffalo and cattle have similar DNA sequence (although they have different polymorphisms), the conventional cattle (UMD 3.1) and most studied assembly used for buffalo genomic analysis (de Camargo *et al.* 2015; Iamartino *et al.* 2017) was used for SNP-linkage disequilibrium-based chromosomal rearrangement and compared with the new buffalo genome assembly (UAO_WB_1). Only the markers with known position referenced in UMD3.1 autosomes were used. The data consisted of the same 46,378 SNPs located in UMD3.1 and described previously, from which 43,697 SNPs also had known position (probe markers aligned against reference genome) in UAO_WB_1. The five submetacentric chromosome had 16,124 and 15,233 SNPs in UMD3.1 and UAO_WB_1 respectively. The Spearman correlation and the linear regression coefficients between the rearranged SNPs position and UAO_WB_1 assembly were estimated. The number of possible misplaced SNPs, using the approach described by Utsunomiya *et al.* (2016) as well as the LD decay, were also performed using both assemblies.

### Haplotype diversity

The haplotype diversity was used to compare the preservation of the centromeric regions in relation to the other regions of the five first chromosomes (submetacentric) of the water buffalo. Only the Affymetrix panel (highest number of markers) was used for this analysis, considering the same set of markers used for estimating linkage disequilibrium. The Beagle software v.4 (Browning and Browning 2007) was used to estimate the haplotype phases for chromosomes. Sliding windows of 1 Mb, with 0.5 Mb overlapping, were used to obtain the haplotype blocks across the genome. The diversity estimate of these blocks was calculated as $\hat{H}=\frac{N-1}{N}(1-\sum_{i=1}^{l}p_{i}^{2})$ for each haplotype with p_i_ frequency, considering a sample size of N individuals (Nei and Tajima 1981).

### Imputation accuracy

The chromosomal rearrangements were evaluated using the imputation accuracy for buffalo submetacentric chromosomes. It verified the construction of the haplotype phases by comparing the UAO_WB_1 assembly to the chromosomal structures (arms) based on UMD3.1 with and without rearrangements. If the rearrangement was inadequate, it is expected that the imputation accuracy in centromeric regions decrease compared to the non-rearranged. For this analysis, only the Affymetrix panel was used, with the same set of markers used for LD estimation.

The imputation was performed using Beagle software v.4 (Browning and Browning 2007). Scenarios with 10% of markers (90% omitted), 15%, 20%, 25% and 30% of markers were considered in order to calculate the imputation accuracy given by allelic correlation (r^2^a) between true and imputed genotypes, and by the proportion of correctly imputed alleles (PERC). The markers were omitted randomly and equidistantly in relation to their order. The analyses were performed by randomly partitioning the data into reference and imputation sets, represented by 262 and 87 animals, respectively. Cross-validation was performed on a fourfold scheme. Accuracy measurements of centromeric regions were compared using UAO_WB_1 and structures with and without rearrangement by F-test and Tukey multiple comparisons of means test (*P* < 0.05).

### Data availability

All bovids genotypic data are available in the public WIDDE database (Sempéré *et al.* 2015). Supplemental material available at figshare: https://doi.org/10.25387/g3.11994495.

## Results

### Genetic clusters

**Figure 1:**
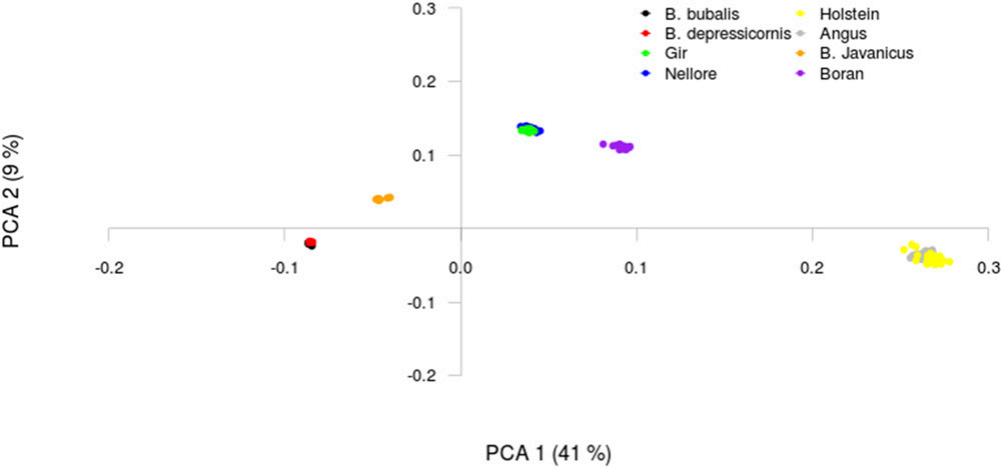
Genetic clusters resulting from the two main principal components (PCA) of the genomic relationship matrix among the studied bovids species. This analysis considered 33,198 markers.

The principal component analysis (PCA) of the genomic relationship matrix separated the data into five different clusters (Figure 1). *Bos taurus taurus* breeds and buffalo species were grouped in single clusters, which were completely separated by component 1. *Bos taurus indicus* was discriminated in two clusters: Indian (Nellore and Gir) and African (Boran) breeds. *Bos javanicus* was closer to the buffalo cluster than to the *Bos taurus*. Zebu breeds, especially the Indian breeds, were closer to both buffalo and *Bos javanicus* than to the European breeds.

**Figure 2:**
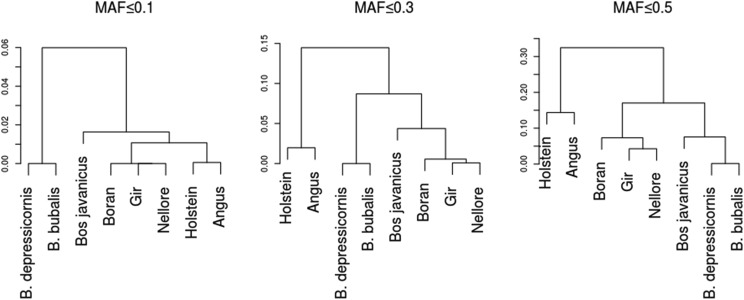
Genetic clusters obtained by Ward’s hierarchical method from the Jaccard (dis)similarity matrix among the highest frequency alleles of each population, for MAF ≤ 0.5 (26,063 SNPs), MAF ≤ 0.3 (8,391 SNPs), MAF ≤0.1 (2,152 SNPs).

The hierarchical grouping method (HC) allowed quantifying the distances among species/breeds (Figure 2). The results were similar to those obtained with PCA for markers with MAF up to 0.5 (HC0.5). Two larger clusters were observed, one grouped European animals and the other, tropical animals. Within the tropical animals, a cluster formed by *Bos taurus indicus* was divided into Indian and African animals, and another cluster was formed by other species, with subdivision in *Bos javanicus* and buffaloes.

For MAF lower than or equal to 0.3 (HC0.3) most of the clusters remained the same as above, except for the tropical animal intermediate cluster where the subgroups were differentiated between the genera *Bos* and *Bubalus*. For MAF smaller than 0.1 (HC0.1), the clusters were consistent with the zoological classification, the genera *Bos* and *Bubalus* were first discriminated, followed by the species *Bos javanicus* and *Bos taurus* classified into distinct subgroups. The *Bos taurus* was subdivided into *taurus* and *indicus*, highlighting a division between African and Asian breeds.

### Introgression analyses

**Figure 3:**
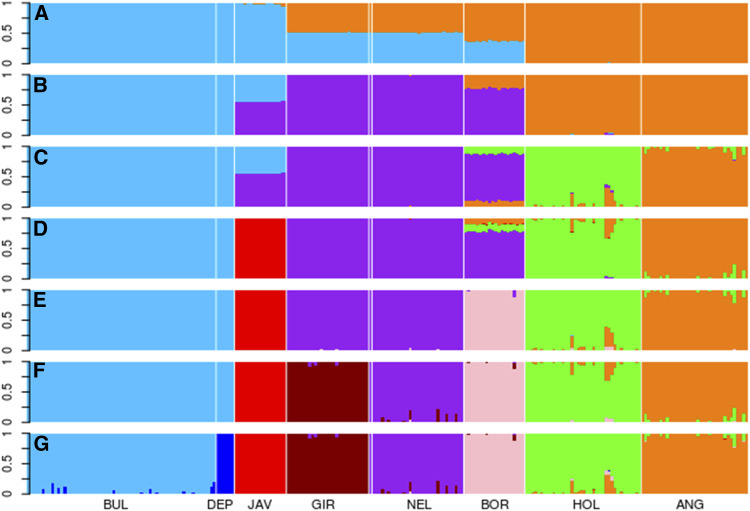
Composition of breeds and species by animal considering different numbers of genetic clusters (k = 2 to 8, A to G, respectively). The analyzed populations were *B. bubalis* (BUL), *B. depressicornis* (DEP), *B. javanicus* (JAV), Gir (GIR), Nellore (NEL), Boran (BOR), Holstein (HOL), and Angus (ANG). 33,198 markers were used in the analysis.

In the introgression analysis, the cluster number is defined *a priori*, and for each individual the proportion of each cluster is discriminated. When the number of clusters was 2, the buffaloes and the *Bos javanicus* were classified in the same cluster while *Bos taurus taurus* was in another genetic cluster (Figure 3). Among the zebus (*Bos taurus indicus*), the Boran (African) had a greater participation in *Bos taurus taurus* cluster, while the Indian breeds had approximately 50% in this cluster and 50% in buffalo cluster.

When the number of clusters were three, a third group of zebus was distinguished. The Boran animals presented introgressions of the taurine group. *Bos javanicus* was partially from the buffalo group and most from Zebu group. When the number of clusters was 4, Holstein was differentiated from Angus and the taurine introgression observed in Boran was subdivided between these breeds. When the number of clusters increased to 5 and 6, *Bos javanicus* and *Boran* were separated respectively. The distinction between Nellore and Gir occurred only when the number of clusters was equal or greater than 7.

The last group to distinguish was the *B. bubalis* and *B. depressicornis* species when the number of clusters was greater than or equal to 8. A low degree of *B. depressicornis* introgression was observed in *B. Bubalis* for this number of clusters while no other introgressions were observed among genera.

### Linkage disequilibrium and chromosomal rearrangement

**Figure 4:**
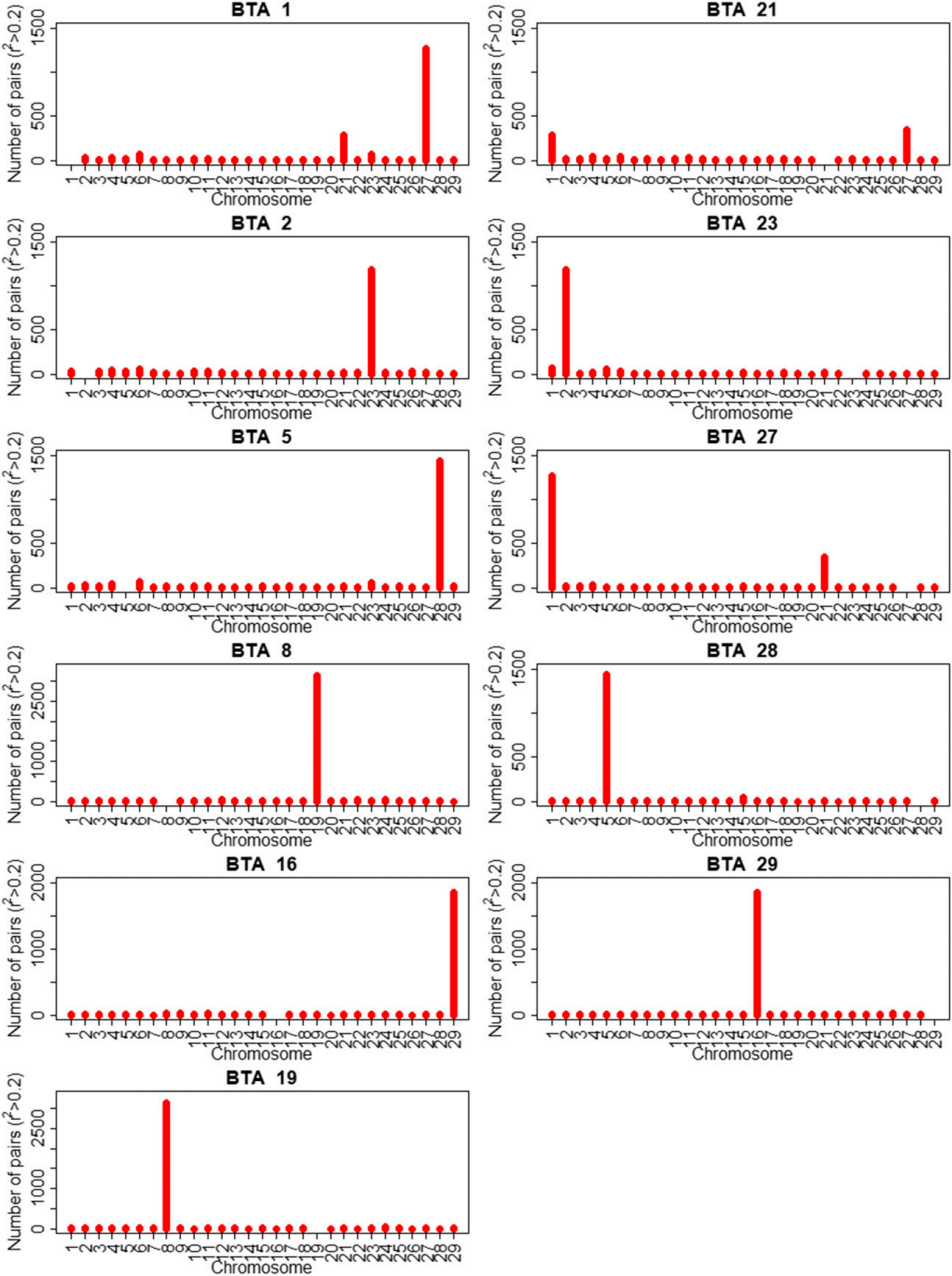
Number of SNPs pairs with r^2^ > 0.20 between the chromosomes with bovine reference (BTAs) in water buffaloes obtained with the specific Buffalo panel. Only chromosomes which had high number of SNPs pairs with r^2^ > 0.20 against others were plotted.

Figure 4 presents the number of SNP pairs with r^2^> 0.20 among all chromosomes and the chromosomal clusters. As expected, the chromosomes that shared the highest number of markers in high LD were 1 and 27 (230 by Affymetrix and 60 by Illumina); 2 and 23 (232 by Affymetrix and 47 by Illumina); 5 and 28 (199 by Affymetrix and 49 by Illumina); 8 and 19 (307 by Affymetrix and 64 by Illumina); and 16 and 29 (303 by Affymetrix and 80 by Illumina). Linkage disequilibrium between pairs of chromosomes with the cattle genome as reference are regions close to the centromeres in the cattle chromosomes, considering their acrocentric forms. These regions also correspond to the beginning of base pair counts of the chromosomes by the bovine assembly reference (Bovine Genome Sequencing and Analysis Consortium 2009; Zimin *et al.* 2009). The results with the Affymetrix panel also indicated that the segments between 59,800,000 bp and 60,500,000 bp of BTA 21 are highly related to the disequilibrium region between chromosomes 1 and 27 (Figure 4).

**Figure 5:**
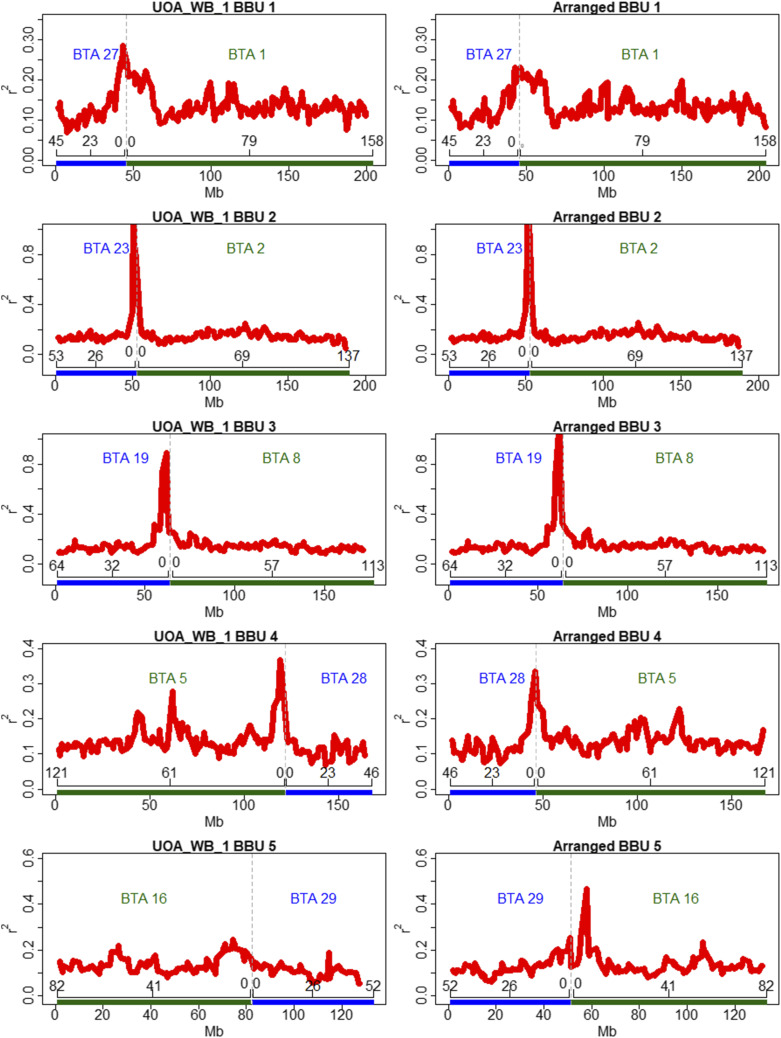
Estimates of linkage disequilibrium (r^2^ - y-axis) for 4 Mb windows along the top five water buffalo chromosomes (BBUs) in megabases (Mb – x-axis), using UOA_WB_1 (left side - UOA_WB_1 on the top) and UMD3.1 rearranged for buffalo chromosomes (right side - Arranged on the top). Inside each figure, on the bottom (x-axis) there is a reference to the cattle chromosomes (BTAs) also in megabases.

Subsequently, the grouped chromosome structures (part of submetacentric buffalo chromosome) could be rearranged to the initial ends parts of the whole bufallo chromosomes, according with number of SNP pairs in high LD. The approximation for a new genomic coordinate for these SNPs in buffaloes chromosomes can be obtained from bovine reference (BTA) as follows: shorter BTA (shorter arm of buffalo chromosome) was given as [(shorter BTA length – SNP position in shorter BTA)] and larger BTA length as [shorter BTA length + (larger BTA length – SNP position in larger BTA)]. This arrangement allowed to observe a higher LD level in the regions near to the chromosome centromeres (Figure 5). No regions with intense LD depression were found.

Higher LD levels were observed for both BBU2 and BBU3 centromeres. However, BBU3 had a greater area of LD extension in the pericentromeric region (base of the higher peak) than BBU2. The BBU4 and BBU5 presented intermediate LD levels, and the highest LD level of BBU5 was observed in the pericentromeric region. The lowest LD level in the centromeric region was observed in BBU1. It also exhibited a greater extension of high LD in comparison to other chromosomal regions. Only the BBU5 assembled with UAO_WB_1 did not show the high LD expected by the rearrangement in the centromere region, indicating the suitability of our rearrangement as model for study the arms and centromere in buffalo submetacentric chromosomes.

### Genome assemblies and misassembly signature

**Figure 6:**
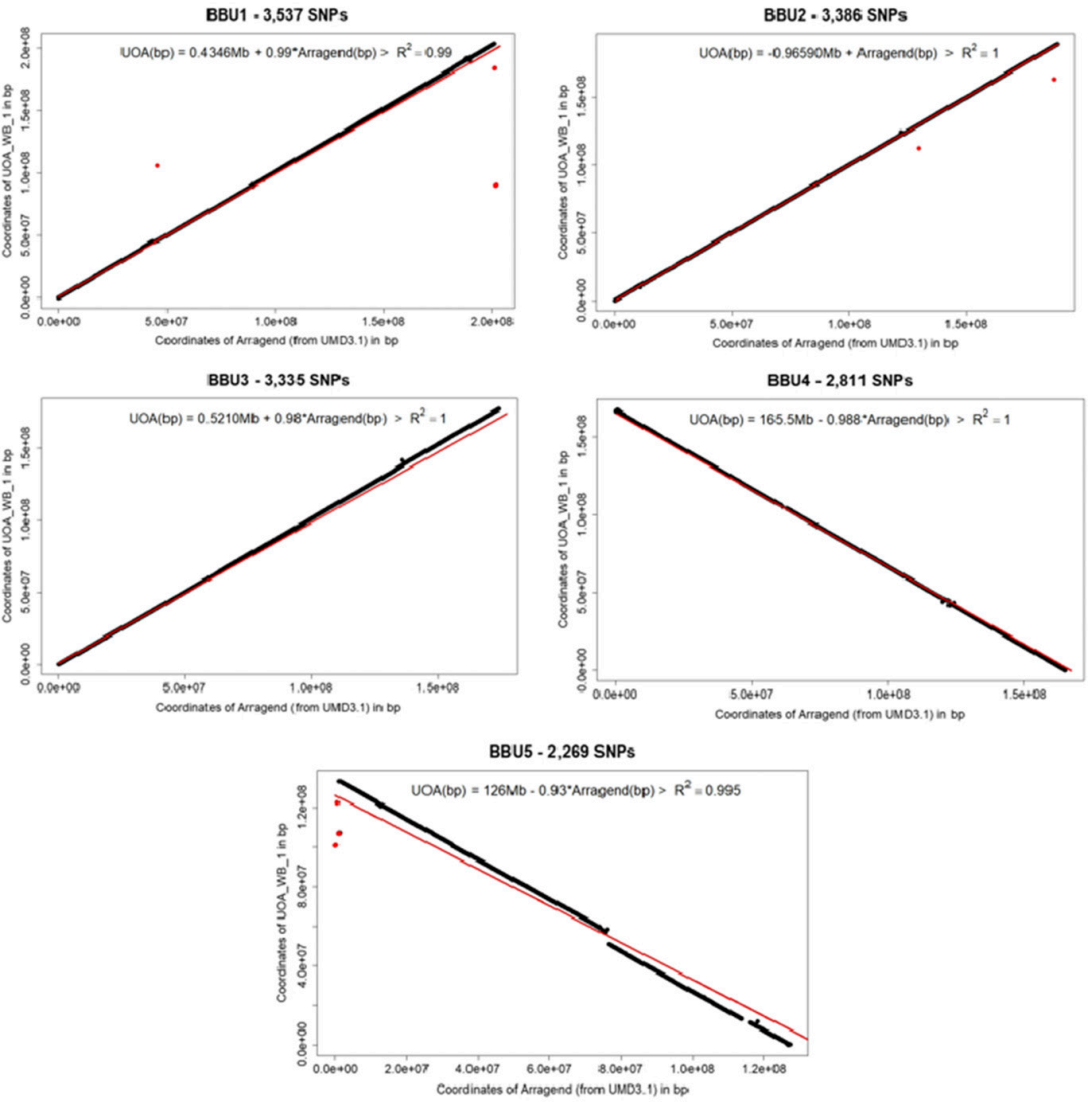
Plot of the coordinates in base pair (bp) of UOA_WB_1 (y - axis) and UMD3.1 rearranged for buffalo chromosomes (Arranged) assemblies (x -axis) of the five first buffalo chromosomes. The red line represents the expected trend. Inside each figure, on the top, there is a linear regression equation of the buffalo genome coordinate (UAO) in base pair according to the rearranged coordinates from cattle genome (Arranged) and its coefficient of determination (R^2^).

The modulo of the value of the Spearman’s rank correlation coefficient (not shown) and coefficient of determination of the linear regression (Figure 6) using a common subset of SNPs for the five submetacentric between the two genome assemblies were close to 1, except for the BBU1, which showed coefficients > 0.99. Negative slope coefficients were observed for BBU4 and BBU5 showing that the position of these chromosomes were oriented from largest to the shortest arm in UAO_WB_1 assembly, differently of the BBU1, BBU2 and BBU3. The axes of the assemblies per chromosome in Figure 6 showed high agreement for chromosome length between the rearrangement and UAO_WB_1 assembly, as well as the small bias observed in the module intercept of the first three chromosomes (< 1 Mb). All markers far from the expected positions in the graphics (18 in BBU1; 3 in BBU2; and 17 in BBU5) were in genomic regions previously reported by Utsunomiya *et al.* (2016) as misassembled in UMD3.1

**Figure 7:**
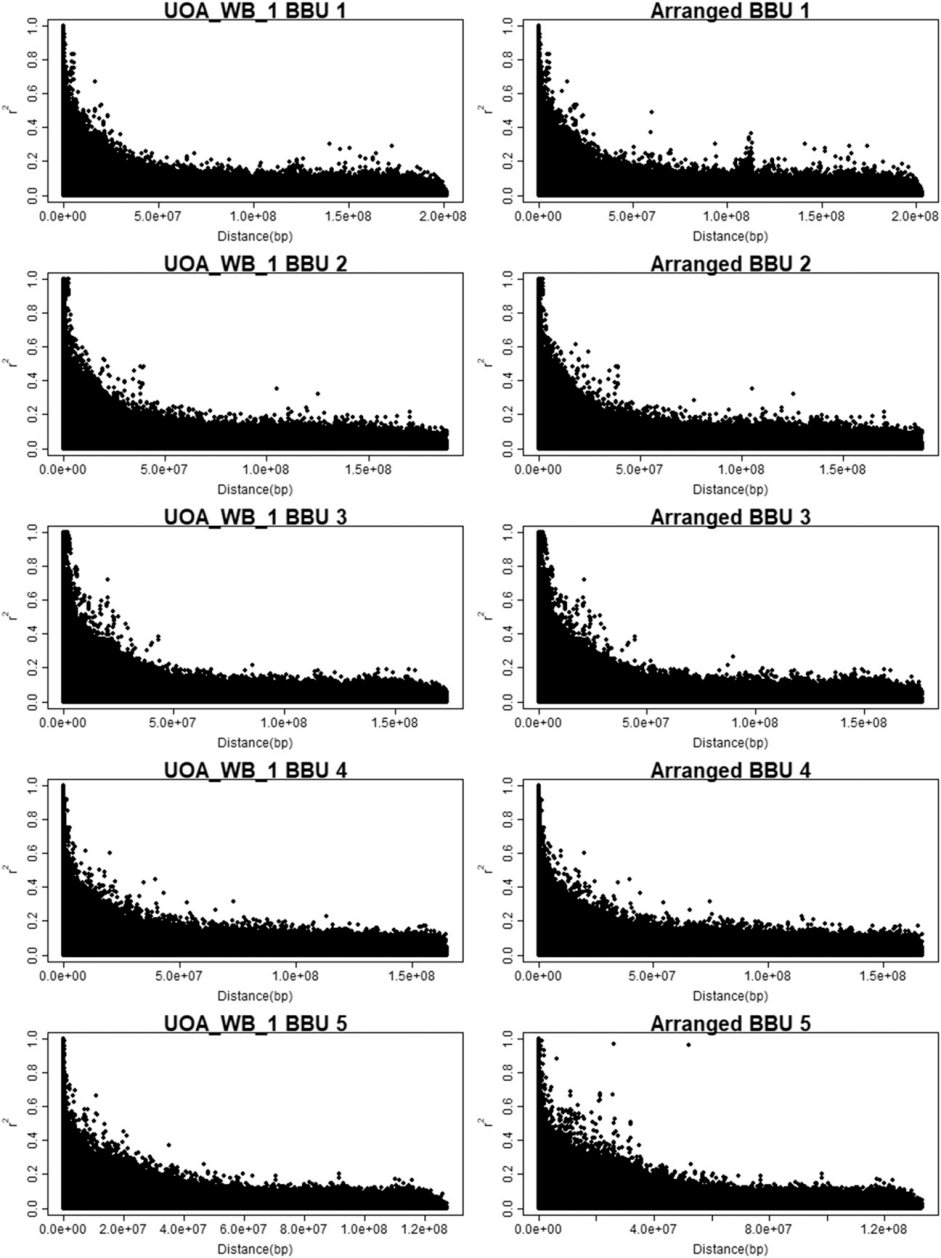
Linkage disequilibrium (r^2^ - y-axis) decay along the physical distance (bp) of the five first water buffalo chromosomes (BBUs) in megabases (Mb – x-axis), using UOA_WB_1 (left side - UOA_WB_1 on the top) and UMD3.1 rearranged for buffalo chromosomes (right side - Arranged on the top).

As expected, all SNPs detected as possible misplaced in the UAO_WB_1 were also detected in the rearrangement assembly, except the SNP in 79,378,396 on BBU5, as well as a lower number of possible misplaced SNPs (Supplemental Table 1; Figure 7). The LD decay presented similar graphics between rearrangement and UAO_WB_1 assembly, except for the BBU1 and BBU5, which present 2 extra hotspot high LD in long distances in our rearrangement. By the arrangement, the BBU4 had lowest number of possible misplaced SNP detected intra/inter chromosome and the BBU5 the greatest number, while for UAO_WB_1 the BBU4 and BBU3 had the lowest and greatest, respectively. Most part of the SNPs detected using the rearrangement were not in UMD3.1 misassemble regions, however some markers, such as four consecutive SNPs on BBU5 (BTA16:70,856,535-70,919,182) were (Utsunomiya *et al.* 2016).

### Haplotype diversity

**Figure 8:**
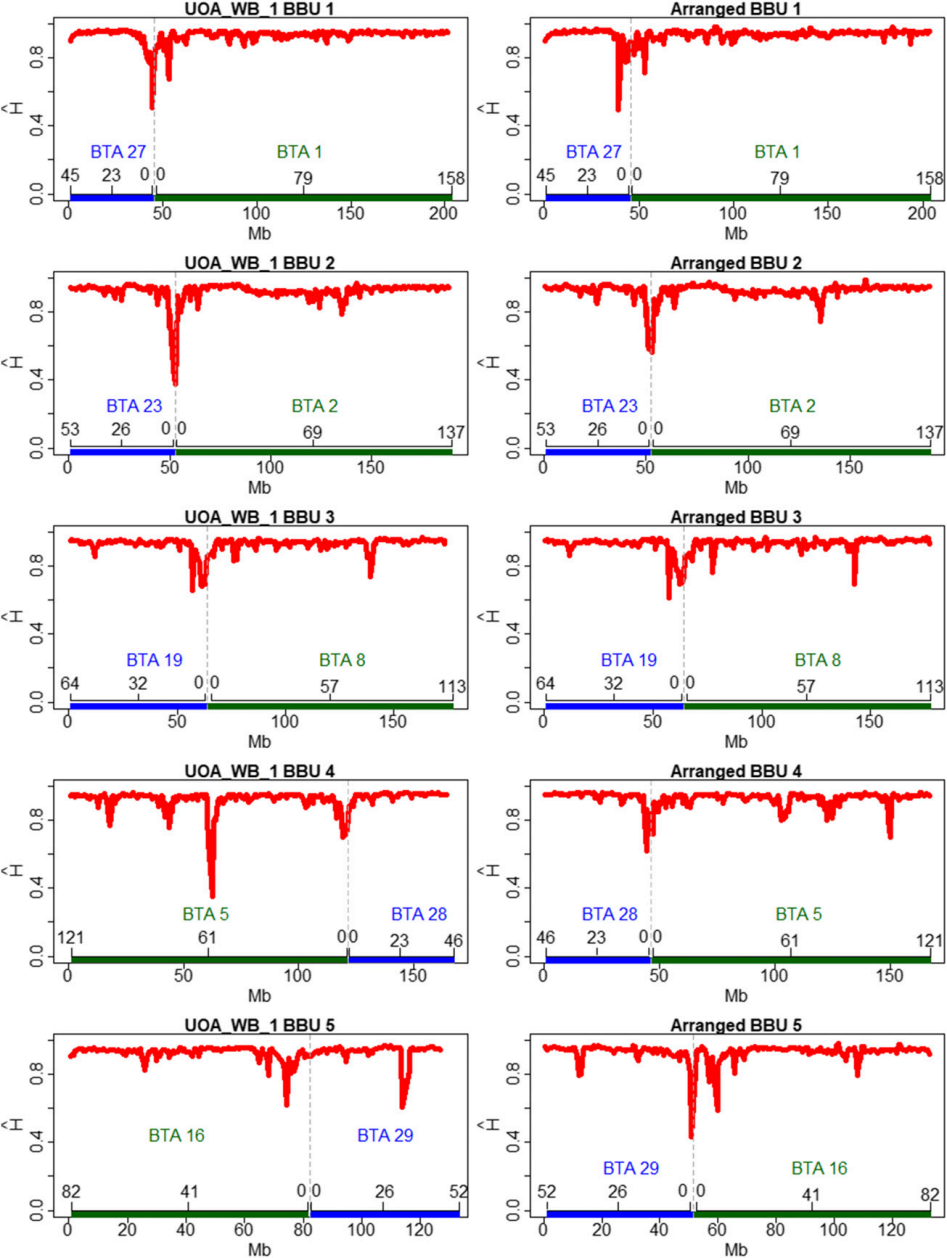
Estimates of haplotype diversity ($\hat{H}$) for 1 Mb windows along the five first water buffalo chromosomes (BBUs), using UOA_WB_1 (left side - UOA_WB_1 on the top) and UMD3.1 rearranged for buffalo chromosomes (right side - Arranged on the top). Inside each figure, on the bottom (x-axis), there is a reference to the cattle chromosomes (BTAs) also in megabases.

Estimates of haplotype diversity along the five submetacentric water buffalo chromosomes are presented in Figure 8 using the rearranged and UAO_WB_1 genome assemblies. This figure shows that the centromeric and pericentromeric regions are less diverse than the other chromosomal regions. Loci with less diverse haplotypes were also found in some extra-centromeric regions corresponding to the cattle chromosomes BTA2, BTA8, BTA5, BTA16, and BTA29. However, haplotype diversity levels of extra-centromeric regions were always higher than those observed in the centromeres except for BBU4 and BBU5 assembled with UAO_WB_1. Thus, the rearranged genome cattle assembly was considered more suitable for the haplotype diversity analysis and may be used as an inference model of centromeric regions. The least diverse centromere in the rearranged chromosomes was BBU5, followed by BBU1, BBU2, BBU3 and BBU4. BBU2 and BBU4 had less diverse single peaks located in the exact position estimated to be the centromere of the chromosomes. BBU5 has the first conserved peak in the exact estimated centromeric region, and another one in the pericentromeric region. BBU1 and BBU3 had diffuse conserved peaks, with the highest peaks next to the estimated position of the centromere (pericentromeric region).

### Imputation accuracy

The analysis of the imputation accuracy allowed inference about the quality of the rearrangements of the water buffalo submetacentric chromosomes from bovine reference. When the whole chromosome was considered, the means of the imputation accuracy were similar between the UAO_WB_1, rearranged and non-rearranged chromosomes (Supplemental Table 2 and 3). The accuracies varied according to the scenarios (from 10 to 30%), when r^2^a ranged from 0.87 to 0.91, and PERC, from 0.92 to 0.96. Significant differences in the accuracy values were not observed between UAO_WB_1 and rearranged when the whole chromosome was considered, except for the scenario with 15% of markers for r^2^a in the BBU1, and for PERC in the BBU1 and BBU4 (Supplemental 2 and 3). However, when we considered, a 1 Mb window on both sides of the centromere, significant differences by Tukey (*P* < 0.05) with highest performance by the rearranged assembly were found in the first four chromosomes in at least one scenario (Table 1 and 2). The BBU1 had the highest gain in accuracy in the centromere with the rearrangement, increasing the r^2^a and PERC up to 11% and 9%, respectively comparing to non-rearranged, and 12% and 8% comparing to UAO_WB_1. Using UAO_WB_1, there was a small trend to decrease the imputation accuracy in the centromere according to number of markers increment, indicating the lowest suitability of the UAO_WB_1 for genomic studies in the centromeric regions. For non-rearranged assembly, the means in the centromere were higher for BBU3 in the scenario with 15% of the markers and BBU4 in the 15% and 30% scenarios, with no significant difference from the rearranged assembly. In the most part of the scenarios from the BBU4 and BBU5, the rearranged assembly had the highest performance, with no significant difference as well. However, the dispersion of the values (standard deviation) in the centromere of these chromosome were high. This result indicates that the difference between the scenarios in the BBU4 and BBU5 might be caused by the SNP sampling (scenario’s composition).

**Table 1:** Mean and standard deviation of the allelic correlations between the imputed and true genotypes in the centromeric regions (± 1 Mb of the centromere) for the five *B. bubalis* submetacentric chromosomes, considering the UOA_WB_1 buffalo genome assembly, and the bovine genome assembly (UMD) *a priori* (Non Arranged) and *a posteriori* (Arranged) chromosome arrangement to the imputation in different marker scenarios

CHR	Assembly	SNP Set				
		10%	15%	20%	25%	30%
	UOA_WB_1	**0.949^a^ ± 0.11**	**0.947^a^ ± 0.08**	0.894 ± 0.20	0.870 ± 0.16	**0.844^a^ ± 0.21**
BBU1	Arranged	**0.946^a^ ± 0.11**	**0.961^a^ ± 0.08**	0.959 ± 0.09	0.962 ± 0.09	**0.945^b^ ± 0.12**
	Non Arranged	**0.849^b^ ± 0.20**	**0.846^b^ ± 0.23**	0.862 ± 0.21	0.886 ± 0.22	**0.894^b^ ± 0.15**
	UOA_WB_1	0.975 ± 0.02	**0.931^a^ ± 0.02**	**0.922^a^ ± 0.13**	0.922 ± 0.13	**0.902^a^ ± 0.04**
BBU2	Arranged	0.975 ± 0.02	**0.979^b^ ± 0.02**	**0.982^b^ ± 0.01**	0.982 ± 0.01	**0.978^b^ ± 0.02**
	Non Arranged	0.965 ± 0.03	**0.976^b^ ± 0.02**	**0.982^b^ ± 0.01**	0.982 ± 0.01	**0.971^b^ ± 0.02**
	UOA_WB_1	**0.985^a^ ±0.01**	0.960 ± 0.03	**0.861^a^ ± 0.18**	0.854 ± 0.23	**0.927^a^ ± 0.02**
BBU3	Arranged	**0.986^a^ ±0.01**	0.957 ± 0.11	**0.985^b^ ± 0.01**	0.952 ± 0.13	**0.984^b^ ± 0.01**
	Non Arranged	**0.971^b^ ±0.02**	0.978 ± 0.02	**0.985^b^ ± 0.01**	0.946 ± 0.13	**0.981^b^ ± 0.01**
	UOA_WB_1	0.907 ± 0.20	0.896 ± 0.12	0.918 ± 0.14	0.883 ± 0.12	0.895 ± 0.14
BBU4	Arranged	0.949 ± 0.12	0.950 ± 0.13	0.947 ± 0.13	0.949 ± 0.13	0.906 ± 0.19
	Non Arranged	0.946 ± 0.12	0.952 ± 0.13	0.944 ± 0.14	0.948 ± 0.13	0.944 ± 0.14
	UOA_WB_1	0.958 ± 0.03	0.915 ± 0.03	0.937 ± 0.03	0.775 ± 0.36	0.881 ± 0.03
BBU5	Arranged	0.943 ± 0.15	0.935 ± 0.17	0.939 ± 0.19	0.931 ± 0.19	0.934 ± 0.18
	Non Arranged	0.904 ± 0.16	0.925 ± 0.17	0.935 ± 0.17	0.922 ± 0.18	0.924 ± 0.20

Values in bold presented significant differences (*P* < 0.05) by F-test. Different small letters presented differences (*P* < 0.05) in the mean between the buffalo assembly (UOA_WB_1), arranged and non-arranged chromosome and marker scenario by Tukey multiple comparisons of means test.

**Table 2:** Mean and standard deviation the proportion of alleles correctly imputed in the centromeric regions (± 1 Mb of the centromere) for five *B. bubalis* submetacentric chromosomes, considering the UOA_WB_1 buffalo genome assembly, and the bovine genome assembly (UMD) *a priori* (Non Arranged) and *a posteriori* (Arranged) chromosome arrangement to the imputation in different marker scenarios

CHR	Assembly	SNP Set				
		10%	15%	20%	25%	30%
	UOA_WB_1	**0.975^a^ ± 0.03**	**0.969^a^ ± 0.02**	**0.950^a^ ± 0.06**	**0.929^a^ ± 0.04**	**0.908^a^ ± 0.06**
BBU1	Arranged	**0.973^a^ ± 0.03**	**0.979^a^ ± 0.02**	**0.979^b^ ± 0.02**	**0.981^b^ ± 0.02**	**0.977^b^ ± 0.03**
	Non Arranged	**0.893^b^ ± 0.11**	**0.940^b^ ± 0.06**	**0.943^a^ ± 0.07**	**0.958^c^ ± 0.05**	**0.942^b^ ± 0.07**
	UOA_WB_1	0.981 ± 0.02	**0.945^a^ ± 0.02**	**0.947^a^ ± 0.04**	**0.957^a^ ± 0.03**	**0.927^a^ ± 0.03**
BBU2	Arranged	0.981 ± 0.02	**0.984^b^ ± 0.02**	**0.983^b^ ± 0.02**	**0.988^b^ ± 0.01**	**0.982^b^ ± 0.02**
	Non Arranged	0.974 ± 0.02	**0.982^b^ ± 0.02**	**0.980^b^ ± 0.02**	**0.988^b^ ± 0.01**	**0.978^b^ ± 0.01**
	UOA_WB_1	**0.988^a^ ± 0.01**	0.979 ± 0.01	**0.937^a^ ± 0.05**	**0.936^a^ ± 0.06**	**0.944^a^ ± 0.02**
BBU3	Arranged	**0.989^a^ ± 0.01**	0.982 ± 0.02	**0.988^b^ ± 0.01**	**0.979^b^ ± 0.03**	**0.987^b^ ± 0.01**
	Non Arranged	**0.976^b^ ± 0.01**	0.982 ± 0.01	**0.988^b^ ± 0.01**	**0.975^b^ ± 0.03**	**0.985^b^ ± 0.01**
	UOA_WB_1	0.954 ± 0.07	**0.933^a^ ± 0.03**	0.953 ± 0.04	**0.924^a^ ± 0.04**	0.931 ± 0.04
BBU4	Arranged	0.970 ± 0.05	**0.974^b^ ± 0.03**	0.973 ± 0.04	**0.975^b^ ± 0.04**	0.963 ± 0.06
	Non Arranged	0.967 ± 0.05	**0.976^b^ ± 0.03**	0.970 ± 0.04	**0.973^b^ ± 0.04**	0.974 ± 0.04
	UOA_WB_1	0.969 ± 0.02	0.956 ± 0.01	0.950 ± 0.02	0.897 ± 0.01	0.909 ± 0.04
BBU5	Arranged	0.975 ± 0.05	0.972 ± 0.05	0.976 ± 0.06	0.972 ± 0.06	0.976 ± 0.05
	Non Arranged	0.950 ± 0.04	0.967 ± 0.05	0.972 ± 0.05	0.967 ± 0.05	0.970 ± 0.05

Values in bold presented significant differences (*P* < 0.05) by F-test. Different small letters presented differences (*P* < 0.05) in the mean between the buffalo assembly (UOA_WB_1), arranged and non-arranged chromosome and marker scenario by Tukey multiple comparisons of means test.

## Discussion

The cattle commercial panel used to genotype buffaloes allowed us to infer the homology and the divergence between the species. However, a clear bias in the comparative analyses between the genetic groups was observed because the commercial panel is composed of polymorphic markers of *B. taurus*, especially with taurine breeds (Illumina BovineHD Genotyping BeadChip Data Sheet). This fact was most evident in the analyses of Hierarchical Clusters, because as the MAF limit values decreased, the bias effect of the selection of markers also decreased, resulting in genetic clustering that coincided with the expected zoological classification between the *Bubalus* and the species of *Bos* genus (Vaughan 1986). The genetic similarity/divergence analyses, in fact, inferred about the distance of *B. taurus taurus* to other bovids and the inference across other groups was prejudiced. For example, PCA distances indicated that *B. taurus indicus* was closer to buffalo than to taurine cattle breeds due to marker selection bias. A similar effect was found in cervidae genotyped with cattle SNPs, indicating that *Bos taurus indicus* were closer to cervids than to *Bos taurus taurus* (Kasarda *et al.* 2015). Taurine was used as reference to avoid erroneous interpretations of the results, so the distances were taken from them, not between other groups. However, PC1 fully discriminated buffalo group from the taurine group, so buffaloes can also be considered a reference for comparison (similarity of the genetic groups from buffaloes). It implies that the interpretation of results apart from buffaloes contains little bias and it can be considered valid. It is an important result for the forthcoming chromosomal rearrangements proposed.

The PCA allowed us to observe the discrimination within the genus *Bos* and between the genera *Bos* and *Bubalus*, but not the discrimination between *B. depressicornis* and *Bubalus*. Moreover, when *Bos taurus taurus* was assumed as a reference for comparison, the furthest group were the buffalo, followed by *Bos javanicus* and the Asian *B. taurus indicus* and, finally, the African breed. Hierarchical Clusters (Figure 2 - HC0.5 and HC0.3), and admixture analysis (Figure 3 - k = 2) revealed greater genomic similarities between buffaloes and Asian cattle than to European cattle. These results indicate that buffaloes may actually be closer to *B. javanicus* followed by *B. taurus indicus* and *Bos taurus taurus*. This relation can be attributed to the analogous evolution process of these groups, such as the adaptation of the cattle and buffalo to the same Indian environment (Hoffpauir 1982).

The geographical origins of buffaloes and *B. javanicus* could contribute to explaining the genetic similarity because both of them originated in South-East Asia (Lau *et al.* 1998; Ropiquet *et al.* 2008). After its origins in Southeast Asia, buffaloes spread to northern China and western India, the natural habitat of zebu (Lau *et al.* 1998). Colli *et al.* (2018) suggest that two independent domestication events occurred in Indo-Pakistani region for water buffalo and close to the China/Indochina border for swamp buffalo. However, there are two current explanations of zebu origins in India (MacHugh *et al.* 1997). The first has Auroque (*Bos primigenius primigenius*) as a common ancestor, which despite being Asian, it first originated *Bos taurus* in Europe and after, it diversified into the zebu in India. The second and the most accepted is that zebu developed independently in South Asia from another subspecies of the Auroque, the *Bos primigenius namadicus*.

The high r^2^ and the low haplotypic diversity observed in the centromeric regions of buffalo submetacentric chromosomes are strong evidence of a fusion between the chromosomes of a common ancestor that had 29 acrocentric autosomes (2n = 60), and not a rupture of the submetacentric chromosomes in this supposed ancestral to originate the 29 acrocentric chromosomes (Iannuzzi *et al.* 2009). The hypothesis of a presumed ancestral with 29 autosomes and the greater genomic similarity between Asian Bovidae corroborate the theory that the common ancestor to these bovids was possibly *Bos primigenius namadicus*.

Chromosome structural evidence also indicate similarity across species. For example, *B. javanicus* (2n = 56) have two Robertsonian translocations, while buffaloes have 5 to 6 (2n = 50 and 2n = 48) (Iannuzzi *et al.* 2003; Ropiquet *et al.* 2008). Although translocations are different among these species, they always have involved orthologs to BTA 1, 2, 28 and 29 (Ropiquet *et al.* 2008). Since the translocation process always affects the same groups of chromosomes and occurs between centromeres, the predisposition for translocation may not be only random and structural but it may also play a role in their composition, such as the number of tandem repeats of sequences that may cause instability and consequent centromere displacement during meiosis (Berend *et al.* 2003; Purgato *et al.* 2015). Other evidence is that the most common chromosomal aberration in cattle (intraspecific) is the translocation of 1:29 (De Lorenzi *et al.* 2012; Yimer and Rosnina 2014).

In this way, the ancestor 2n = 60 (common to both groups) possibly had variations/mutations that predisposed translocations with a frequency that allowed the appearance of different species by reproductive isolation. Another evidence about the similarity between zebu and buffalos is the presence of acrocentric Y sex chromosome, unlike the European cattle that have metacentric Y sex chromosome (Iannuzzi *et al.* 2001; Yindee *et al.* 2010). Although these results suggest that Asian cattle may have a common origin independent from the European, the possibilities of crossbreeding between the ancestors of these groups at different periods are plausible in both theories, making it more difficult to analysis the evidence.

The similarity between Asian Bovidae observed can also be attributed to natural selection processes that converged to generate similar functions among the species because these are subject to the same tropical environment and this may have favored the predominance of certain alleles (Vickrey *et al.* 2015). In addition, the similarities obtained with higher frequency alleles are also possibly related to the convergence of artificial selection for domestication and production.

The introgression analysis allowed to discriminate between *Bubalus* species and *B. depressicornis*, when the number of clusters was equal to 8, indicating a slight *B. depressicornis* introgression in the water buffalo. This introgression possibly originated from the centromeric regions of the four submetacentric chromosomes common to these species, considering that these regions are more conserved/preserved than other chromosomal regions (Gallagher *et al.* 1999; Schneider *et al.* 2016). The buffaloes and *Bos javanicus* were clustered together when cluster number was equal to 2. Also, the Asian zebu had a greater buffalo and *B. javanicus* introgression when compared to the European animals, unlike the African zebu that presented greater introgression of the European clustering. This introgression may possibly have resulted from the fact that Boran is a recent breed, originated from mixed taurine breeds (Rege 1999). The possible reasons for the Indian zebu to present greater Asian Bovidae introgression are, in addition to crossbreeding, the homology of the species and the possible convergent evolution of these groups as discussed above. *B. javanicus* was the last to differentiate from buffaloes when the number of clusters increased, this similarity was observed in the other analyses. *B. javanicus* also had buffalo introgressions and a slightly higher zebu proportion when the number of clusters was lower than 5.

Decker *et al.* (2014) reported zebu introgression into *B. javanicus* and considered these animals as belonging to the same cluster, in a study considering *B. javanicus*, Eurasian and African *B. taurus*, for cluster number equal to 3. In our study, when the cluster number was greater than or equal to 5, no relevant introgressions were observed between the *Bubalis* and *Bos* genera and the distinction between the taurines breeds was observed when the number of cluster was lower to 4, while more than 5 clusters were necessary to separate zebus. This result strongly indicates that crosses between the ancestors of these Asian Bovidae of both genera occurred, as it was previously considered. In addition, although no algorithm was used to select the best number of clusters, eight clusters (k = 8) showed to be the best option for these analyses, since these correctly discriminated all genetic groups (species and breeds) in consonance to a biological perspective.

The r^2^ levels were similar to reported to buffalo species by Deng *et al.* (2018) and Mokhber *et al.* (2019). The buffalo specific SNP panel enabled a better inference of LD among markers, especially between chromosomes than the one resulted from the cattle SNP panel. The LD between the markers of the homologous structures allowed not only to infer which structures were related such as the FISH (Fluorescent in Situ Hybridization) cytogenetic probes (Iannuzzi *et al.*, 2003), but also to orient their positioning such as in the original buffalo assembly (Low *et al.* 2019), and possible evolution.

The use of LD to verify the relationship of the homologous structures still had the advantage of being easily obtained by panels of markers that are used in large scale for other genomic studies, without the necessity to design specific probes with single functionality. Comparing to sequencing data, the LD approach allows to use many individuals in the analysis at a low-cost; whereas in sequencing, only one or few individuals are used (despite this method allows greater number of variants). The disadvantage is that LD estimates can be affected by several factors such as history and population structure, effective population size, sample size, markers density and distribution, and strict filtering of SNPs (Hayes *et al.*, 2003; Yan *et al.*, 2009; Bohmanova *et al.*, 2010).

LD methodology also detected a correlation of a 0.7 Mb region of BTA21 with BTA 1 and 27. Although it appears to be a translocation in buffaloes, Utsunomiya *et al.* (2016) reported that this region is a possible assembling error of the UMD3.0 bovine reference.

Our rearrangement showed high agreement with UOA_WB_1 for SNP order and chromosome length according to the correlations and linear regression analyses. These results were expected since, according to Low *et al.* (2019), the UOA_WB_1 was scaffolded in an order conserve synteny with the homologous *Bos taurus* genome (UMD3.1). These authors also observed good agreement for the chromosome sizes and proportion of sequences aligned to corresponding homologous *B. taurus* chromosomes, comparing UOA_WB_1 and UMD3.1.

Some misassembled SNPs comparing UOA_WB_1 and UMD3.1 were previously reported by Utsunomiya *et al.* (2016). Additional possible misassembled SNPs were also detected in our study. Possible misassembled SNPs can be detected in new regions due to the assessment bias of the SNP panels as well as the different polymorphisms in the buffalo genome. In general, all the results confirm that the buffalo genome assembly was more accurate than the rearrangement of the UMD3.1 to predict the right marker position. However, the rearrangement allowed to include many SNPs given as unknown position (1,036) in UOA_WB_1, despite a few of them were considered as potential misplaced SNPs. The main difference between the genome assemblies is not related to segment translocations, but is due to the quality of sequence methods, since the UMD3.1 used Sanger (https://www.ncbi.nlm.nih.gov/assembly/GCF_000003055.6/) and while UOA_WB_1 used PacBio technology (https://www.ncbi.nlm.nih.gov/assembly/GCF_003121395.1/). These results indicate that the improvement in sequence technologies used for cattle as well as new cattle assemblies, would improve the quality of the rearrangement. Although we have not tested, the results also suggest that the improvement in cattle assembly can also help any specific buffalo assemblies, mainly in misassembled regions.

The r^2^ estimates were similar at the telomeres of the five studied chromosomes and regions with intense LD depression were not observed. This result indicated that there were no large deletions (> 4 Mb) in these buffalo chromosomes compared to the cattle homologs, as was observed by Low *et al.* (2019) with sequence data. However, significant differences were observed when comparing the centromeric and telomeric regions. It indicates a fusion process in common ancestors to originate buffalo submetacentric chromosomes, instead of the rupture of the ancestral chromosomes to generate the acrocentric chromosomes in cattle. The BBU5 assembled with UAO_WB_1 had no high LD in the centromere region, as we observed in our rearrangement. This high LD in the expected region indicates that the rearrangement is the better way to study LD levels in the submetacentric chromosomes.

High LD levels in the centromeric and pericentromeric regions have been observed in plant and animal species (Smith *et al.* 2005; Shi *et al.* 2010; Talbert and Henikoff 2010). The possible explanation is that the probability of crossing-over is very low in these regions or that there is no crossing-over in the centromere (Shi *et al.* 2010; Talbert and Henikoff 2010). Higher LD levels and less diversity were observed in the aforementioned studies, when compared to the present study. It possibly happens because the translocations in buffaloes are more recent. The species has only five submetacentric chromosomes, while the other species have all the autosomes as acrocentrics (Smith *et al.* 2005; Shi *et al.* 2010). Therefore, the low recombination rate and the reduced diversity in the centromere may serve as indicators of the previous translocation processes.

The different LD patterns observed in the centromeres of the buffalo submetracentric chromosomes may be due to the order of occurrence of the translocations during evolution time. The ascertainment bias of the markers in this region and the current location of the centromere may also interfere in the LD behavior and haplotype diversity. The ascertainment bias effect was minimized by adjusting the physical distance, using a LD decay function. Five LD peak patterns were observed on the submetacentric chromosomes of water buffaloes:

1. High disequilibrium (r^2^ ≈1) in the centromeric region and small LD extension in the pericentromeric region (BBU 2);
2. High disequilibrium in the centromeric region (r^2^ ≈1) and slightly higher LD extension in the pericentromeric region (BBU 3);
3. Medium disequilibrium in the centromeric region (r^2^ ≈0.4) and greater LD extension region (BBU 4);
4. Low disequilibrium (r^2^ ≈0.2) and high extension of the LD region (BBU 1);
5. Extension of LD very high in the pericentromeric region and, medium, in the centromeric region (BBU 5).

**Figure 9:**
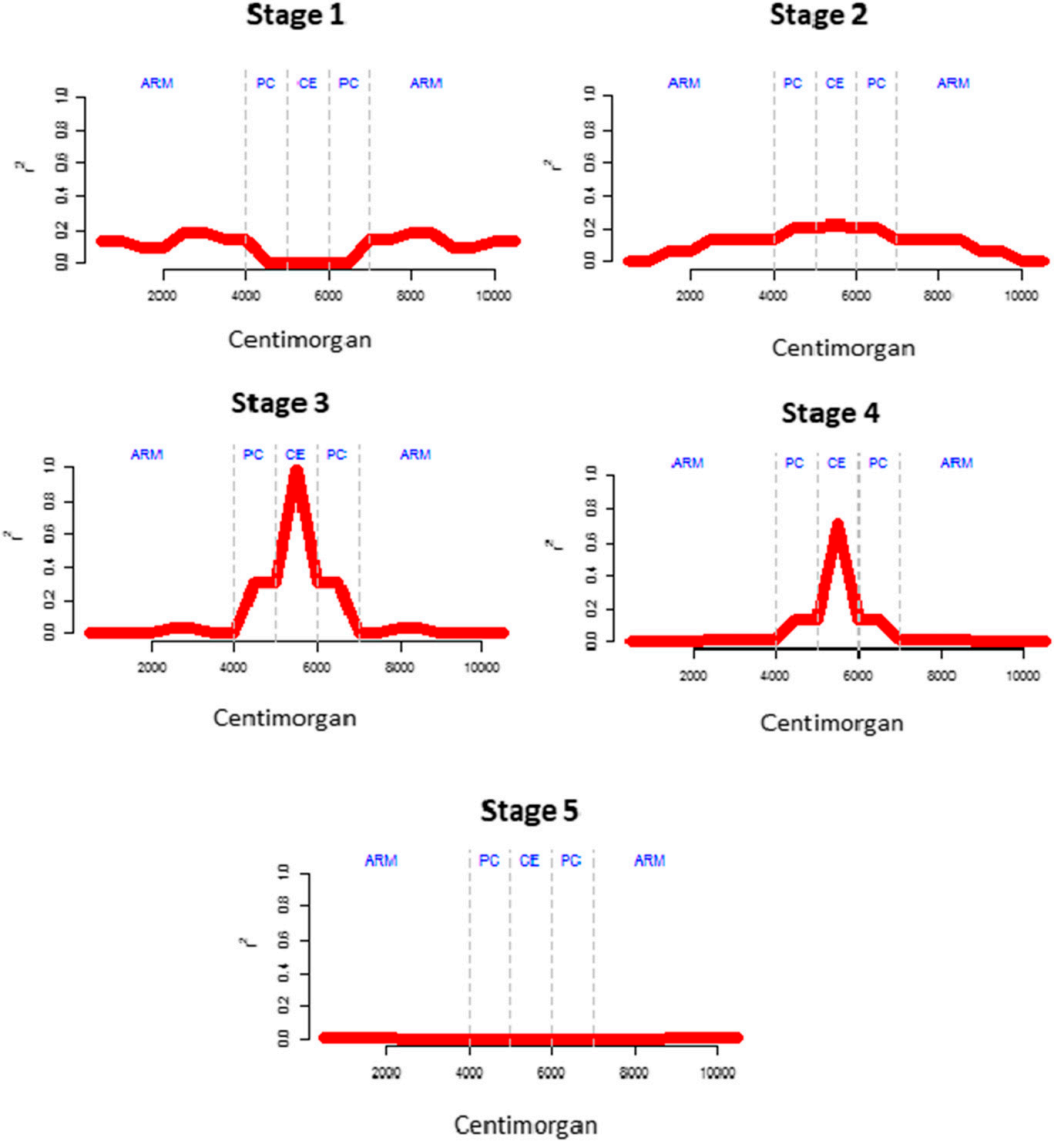
Hypothetical model on linkage disequilibrium (r^2^) level behavior in chromosome arms (ARM), pericentromeric (PE) and centromeric (CE) regions throughout generations after the translocation process forms a new chromosome. The linkage disequilibrium standards were not observed in stages 1 and 5.

Considering that, possibly, the last chromosome to be formed was BBU5, because it is the only one of the 5 submetacentric of *B. bubalis* that is missing in *B. depressicornis* (Gallagher *et al.* 1999), the LD extension in the centromeric region may be related to recent events in these regions. Likewise, Hayes *et al.* (Hayes *et al.* 2003) also correlated the high extension LD between markers to recent events in the history of a population. Therefore, the chronological order of the translocation events was possibly BBU2, BBU3, BBU4, BBU1, and BBU5. Thus, a hypothetical model on the evolution stages was assumed on the LD behavior and diversity in the chromosome arms, pericentromeric and centromeric regions (Figure 9).

Initially, the r^2^ peak tends to be low in the centromeric regions due to the low divergence in this region, increasing over the generations with the increase in diversity (stages 1 and 2). After increasing the diversity in the region, LD (stage 3) also increases. Subsequently, the loci tend to enter in equilibrium over generations, thus decreasing the LD, but much slower than the other chromosomal regions. This equilibrium starts from the outermost centromeric regions toward the center (stage 4). At the end, the low recombination rate of the centromere is balanced with the level of diversity and the region has higher averages than the other chromosome regions, however at a below level of the previous stage (stage 5).

This sequence was conceived without considering the events of genetic bottlenecks that tend to increase LD in both regions, which tend to be dissipated more quickly in the regions away from the centromeres.

Diversity is fundamental to obtain LD estimates while diversity is propagated in the population through recombination. After the occurrence of a translocation, the telomeres of the first new submetacentric chromosome (in the first individual) can still suffer crossover with the non-translocated homologous chromosomes, although at a much lower rate, while the centromere remains with diversity and r^2^ equal to 0. The possibility of reproduction among buffalo subspecies is indicative of this effect. After mutations in the centromere regions (diversity), the estimated LD is already higher due to the lower recombination rate.

The diversity analysis showed that BBU5 had less diversified pericentromeric and centromeric regions besides a greater pericentromeric extension of this conservation. BBU1 also presented a similar profile for these regions, however with larger magnitudes for haplotype diversity. These characteristics also indicate that these chromosomes were the last to be fused, agreeing with the results observed in the LD analyses. With UAO_WB_1 we observed the lowest haplotype diversity levels in extra-centromeric regions for BBU4 and BBU5. Thus, the rearranged genome cattle assembly was considered more accurate and used as model to elucidate evolution process in the centromeric regions.

The r^2^a and PERC values obtained for the buffalo submetacentric chromosomes were close to the variation obtained for Holstein and Angus (Mulder *et al.* 2012; Berry *et al.* 2014) and for Nellore and Gir (Carvalheiro *et al.* 2014; Boison *et al.* 2015), for imputations using 10% of the density of the original marker. This result indicated that the cattle reference for arranging the markers for each orthologous structure could also be used for the buffalo genome. In fact, imputation accuracy is not expected to increase significantly by the rearrangement of the adjacent regions, but to decrease abruptly when the rearrangement is inadequate, reflecting the effect of the misplaced position on the formation of the haplotype blocks (Utsunomiya *et al.* 2016).

Thus, BBU 1 was observed to have the greatest increase in imputation accuracy, especially by correcting the bovine reference between the BTA 21 and BTA27 regions. Regarding the other chromosomes studied, a marked reduction of the imputation accuracy in the centromeric and pericentromeric regions would be observed if the structures had been rearranged inadequately. However, a slight increase in accuracy was observed, indicating a small improvement in the construction of haplotype blocks. This result indicated that the proposed rearrangement was adequate and could be used as the approximate genomic reference for buffalo in studies that are dependent on this coordinates. The better performance of the rearrangements when compared to UAO_WB_1 in many scenarios as well as the small trend of this assembly to decrease the imputation accuracy, according to the number of markers, indicated that rearrangement is the best option for studies in the centromeres, in agreement to the LD and haplotype diversity scan analysis.

The water buffalo has genomic homology to cattle, and its five submetacentric chromosomes come from chromosomal fusions of a common ancestor closer to Asian Bovidae than to taurine. This genomic homology between the species enabled us to use the reference cattle assembly to recreate a buffalo genomic reference by rearranging the submetacentric chromosomes. When using the bovine genomic reference, the rearrangement of the buffalo submetacentric chromosomes could be done by SNP BTA calculations. The centromere of these rearranged chromosomes had the expected profile for the centromere of submetacentric chromosomes exhibiting high linkage disequilibrium and low haplotype diversity, enabling us to hypothesize about evolutionary-genetic events. The imputation accuracy revealed that the proposed rearrangement for these chromosomes was adequate and that the approximate genomic reference for water buffalo can be used in scientific studies. The SNP-based approach is a cheap and easy solution to transfer any cattle assembly technology to buffalo, beyond to help place and order many unplaced SNP/segments in a buffalo assembly. Moreover, the proposed approach can be applied to elucidate other chromosomal rearrangement events in other species and possibly better understand the evolution relationship among them.
